# Supplementary material for: Rapid concerted switching of the neural code in the inferotemporal cortex
Source: Nature. 2026 Mar 25;653(8113):146–55. doi: 10.1038/s41586-026-10267-3 (PMC13148990; doi:10.1038/s41586-026-10267-3)
Supplement: Supplementary file 1 — Reporting Summary [file 41586_2026_10267_MOESM1_ESM.pdf]

nature portfolio

Corresponding author(s): Doris Y. Tsao

Last updated by author(s): 11/25/2025

Reporting Summary

Nature Portfolio wishes to improve the reproducibility of the work that we publish. This form provides structure for consistency and transparency in reporting. For further information on Nature Portfolio policies, see our Editorial Policies and the Editorial Policy Checklist.

Statistics

For all statistical analyses, confirm that the following items are present in the figure legend, table legend, main text, or Methods section.

n/a	Confirmed	
☐	☒	The exact sample size (*n*) for each experimental group/condition, given as a discrete number and unit of measurement
☐	☒	A statement on whether measurements were taken from distinct samples or whether the same sample was measured repeatedly
☐	☒	The statistical test(s) used AND whether they are one- or two-sided<br>*Only common tests should be described solely by name; describe more complex techniques in the Methods section.*
☐	☒	A description of all covariates tested
☐	☒	A description of any assumptions or corrections, such as tests of normality and adjustment for multiple comparisons
☐	☒	A full description of the statistical parameters including central tendency (e.g. means) or other basic estimates (e.g. regression coefficient) AND variation (e.g. standard deviation) or associated estimates of uncertainty (e.g. confidence intervals)
☐	☒	For null hypothesis testing, the test statistic (e.g. *F*, *t*, *r*) with confidence intervals, effect sizes, degrees of freedom and *P* value noted<br>*Give P values as exact values whenever suitable.*
☒	☐	For Bayesian analysis, information on the choice of priors and Markov chain Monte Carlo settings
☒	☐	For hierarchical and complex designs, identification of the appropriate level for tests and full reporting of outcomes
☐	☒	Estimates of effect sizes (e.g. Cohen's *d*, Pearson's *r*), indicating how they were calculated

Our web collection on statistics for biologists contains articles on many of the points above.

Software and code

Policy information about availability of computer code

Data collection	Electrophysiological signals were collected using NHP Neuropixels probes and recorded with the SpikeGLX system (Release_v20230815-phase30, HHMI/Janelia Research Campus, USA) and OpenEphys (version 0.6.4).
Data analysis	We listed all software used in the experiments and for anlaysis in the Methods section.<br>Functional imaging data are prcoessed with Freesurfer (version 4.7.1) and FSL (version 5.0).<br>Electrophysiological data was spike sorted using Kilosort (version 3 and 4).<br>AlexNet (https://github.com/BVLC/caffe/tree/master/models/bvlc_alexnet original version from Krizhevsky A, Sutskever I, Hinton GE. "ImageNet Classification with Deep Convolutional Neural Networks." Advances in neural information processing systems. 2012.)<br>Custom code written in Python 3.10, MATLAB R2023a was used for analysis, which is available from the lead corresponding author upon. reasonable request.

For manuscripts utilizing custom algorithms or software that are central to the research but not yet described in published literature, software must be made available to editors and reviewers. We strongly encourage code deposition in a community repository (e.g. GitHub). See the Nature Portfolio guidelines for submitting code & software for further information.

Data

Policy information about availability of data

All manuscripts must include a data availability statement. This statement should provide the following information, where applicable:

- Accession codes, unique identifiers, or web links for publicly available datasets
- A description of any restrictions on data availability
- For clinical datasets or third party data, please ensure that the statement adheres to our policy

Datasets generated and/or analysed during the current study are available from the corresponding author upon request.

Research involving human participants, their data, or biological material

Policy information about studies with human participants or human data. See also policy information about sex, gender (identity/presentation), and sexual orientation and race, ethnicity and racism.

Reporting on sex and gender	N/A
Reporting on race, ethnicity, or other socially relevant groupings	N/A
Population characteristics	N/A
Recruitment	N/A
Ethics oversight	N/A

Note that full information on the approval of the study protocol must also be provided in the manuscript.

Field-specific reporting

Please select the one below that is the best fit for your research. If you are not sure, read the appropriate sections before making your selection.

☒ Life sciences ☐ Behavioural & social sciences ☐ Ecological, evolutionary & environmental sciences

For a reference copy of the document with all sections, see nature.com/documents/nr-reporting-summary-flat.pdf

Life sciences study design

All studies must disclose on these points even when the disclosure is negative.

Sample size	Sample sizes were maximized given the recording capacity of NHP Neuropixels probes (384 channels) and are consistent with previous studies using similar methods such as She, Liang, et al. "Temporal multiplexing of perception and memory codes in IT cortex" Nature (2024) and Bao, Pinglei, et al. "A map of object space in primate inferotemporal cortex" Nature (2020).
Data exclusions	All neurons encountered along the probe trajectory were recorded. For our main axis mapping analyses, we excluded non-visually responsive cells, non-face-selective cells, and cells that lacked significant axis tuning.
Replication	Results were replicated across at least 2-3 different animals and across 2 different face patches (ML and AM) in independent recording sessions.
Randomization	Stimuli were presented in a randomized order.
Blinding	Investigators were not blinded to experimental groups (face vs non-face object images) because the nature of this study requires separate models to be built for face and object images.

Reporting for specific materials, systems and methods

We require information from authors about some types of materials, experimental systems and methods used in many studies. Here, indicate whether each material, system or method listed is relevant to your study. If you are not sure if a list item applies to your research, read the appropriate section before selecting a response.

Materials & experimental systems

n/a	Involved in the study
☒	☐ Antibodies
☒	☐ Eukaryotic cell lines
☒	☐ Palaeontology and archaeology
☐	☒ Animals and other organisms
☒	☐ Clinical data
☒	☐ Dual use research of concern
☒	☐ Plants

Methods

n/a	Involved in the study
☒	☐ ChIP-seq
☒	☐ Flow cytometry
☐	☒ MRI-based neuroimaging

Animals and other research organisms

Policy information about studies involving animals; ARRIVE guidelines recommended for reporting animal research, and Sex and Gender in Research

Laboratory animals	Three male rhesus macaques (Macaca mulatta) of 8-12 years old were used in this study.
Wild animals	The study did not involve wild animals.
Reporting on sex	Only male animals were used.
Field-collected samples	The study did not involve field-collected samples.
Ethics oversight	All procedures conformed to local and US National Institutes of Health guidelines, including the US National Institutes of Health Guide for Care and Use of Laboratory Animals. All experiments were performed with the approval of the UC Berkeley Animal Care and Use Committee.

Note that full information on the approval of the study protocol must also be provided in the manuscript.

Plants

Seed stocks	*Report on the source of all seed stocks or other plant material used. If applicable, state the seed stock centre and catalogue number. If plant specimens were collected from the field, describe the collection location, date and sampling procedures.*
Novel plant genotypes	*Describe the methods by which all novel plant genotypes were produced. This includes those generated by transgenic approaches, gene editing, chemical/radiation-based mutagenesis and hybridization. For transgenic lines, describe the transformation method, the number of independent lines analyzed and the generation upon which experiments were performed. For gene-edited lines, describe the editor used, the endogenous sequence targeted for editing, the targeting guide RNA sequence (if applicable) and how the editor was applied.*
Authentication	*Describe any authentication procedures for each seed stock used or novel genotype generated. Describe any experiments used to assess the effect of a mutation and, where applicable, how potential secondary effects (e.g. second site T-DNA insertions, mosiacism, off-target gene editing) were examined.*

Magnetic resonance imaging

Experimental design

Design type	Block design
Design specifications	During the fMRI experiment, stimuli were presented in 24 s blocks at an interstimulus interval of 500 ms
Behavioral performance measures	Monkey's eye position was monitored using an infrared eye tracking system (Eyelink). Juice reward was delivered every2–4 s if fixation was properly maintained (within a 3.4 degree square window).

Acquisition

Imaging type(s)	Functional and anatomical imaging
Field strength	3 Tesla
Sequence & imaging parameters	T1-weighted anatomical volumes were measured with MP-RAGE sequence(TR 2,300 ms; IR 1,100 ms; TE 3.37 ms; 0.5mm isotropic voxels) . EPI volumes were acquired in an AC88 gradient insert (Siemens) TR was 2000 ms,TE was 17 ms,voxels were 1 × 1 × 1 mm with an no gap between slices. Matrix size was (96, 96, 64) (read [x], phase [y], slice [z]), thefield of view was 96 × 96 mm in-plane. Flip angle was 80°.
Area of acquisition	Whole brain

Diffusion MRI ☐ Used ☒ Not used

Preprocessing

Preprocessing software	Analysis of functional volumes was performed using the FreeSurfer Functional Analysis Stream(Massachusetts General Hospital). Volumes were corrected for motion and undistorted based on acquired field map.
Normalization	To concatenate different scans, each voxel's responses were percentage transformed with 0 mean value
Normalization template	We did not normalize any imaging data into template. All the analysis were done in the single subject's original space.
Noise and artifact removal	We remove the linear or quadratic trends in the time series.
Volume censoring	Motion noises were removed by putting the motion parameters as the regressors in the GLM analysis.

Statistical modeling & inference

Model type and settings	The analysis used only first-level analysis.
Effect(s) tested	We ran t-tests between different conditions within each single subject.

Specify type of analysis: ☒ Whole brain ☐ ROI-based ☐ Both

Statistic type for inference (See Eklund et al. 2016)	All the analyses were done using voxel-wise inference.
Correction	We did not apply any multiple-comparison correction in the fMRI imaging analysis. We set p value at 0.001.

Models & analysis

n/a	Involved in the study
☒	☐ Functional and/or effective connectivity
☒	☐ Graph analysis
☒	☐ Multivariate modeling or predictive analysis
